# Magnesium calcite in Muschelkalk limestones of the Polish part of the Germanic Basin

**DOI:** 10.1007/s13146-018-0437-y

**Published:** 2018-03-05

**Authors:** Katarzyna J. Stanienda-Pilecki

**Affiliations:** Department of Applied Geology, Faculty of Mining and Geology, Silesian University of Technology, ul. Akademicka 2, 44-100 Gliwice, Poland

**Keywords:** High-Mg calcite, Polish Muschelkalk sediments, Germanic Basin

## Abstract

Magnesium calcite, which is usually observed in many natural low-temperature environments, was identified in Triassic limestones of the Polish part of the Germanic Basin. The occurrence of unstable magnesium calcite in Triassic limestones is remarkable. High-Mg calcite was identified in all beds of the Muschelkalk Karchowice Formation: Lower Crinoidea Beds, Lower Biohermal Beds, Upper Crinoidea Beds and Upper Biohermal Beds. The general chemical formulas of magnesium calcite of mentioned unites are: (Ca_0.87–0.80_,Mg_0.13–0.20_)CO_3_, (Ca_0.83–0.75_,Mg_0.17–0.25_)CO_3_, (Ca_0.81_,Mg_0.19_)CO_3_, (Ca_0.78–0.77_,Mg_0.22–0.23_)CO_3_ respectively. This mineral originated probably during the early stage of diagenesis, during compaction of limestones deposited in a warm, shallow marine environment. Some amounts of magnesium come from high-Mg skeletons of marine organisms (such as echinoderms). Diagnenetic processes and the conditions in seawater, especially higher salinity, influence the preservation of high magnesium calcite in the Muschelkalk limestone of the Polish part of the Germanic Basin.

## Introduction

The area of the Polish part of the Germanic Basin is built of Muschelkalk limestones which include carbonate phases varying in magnesium content. Analyses of low-Mg calcite and high-Mg calcite show that these two carbonate phases differ, in stability, solubility and crystal structure. It is connected with different cationic size of Ca and Mg, ionic radii length and the strength of ionic bonds. The strength of the ionic bond of two Ca ions is higher than the strength of the ionic bond between Ca and Mg ions. Therefore, the structure of a calcite crystal with Mg substitutions has weaker stability than the calcite without substitution. The solubility of high-Mg calcites is the same as that of low-Mg calcite and aragonite at temperatures below 42 °C. Between 25 and 42 °C, calcite with 15 mol% of MgCO_3_ content is stable in relation to low magnesium calcite but unstable with respect to aragonite (Bertram et al. 1991). Calcite with 1.9 mol% amount of MgCO_3_ is stable with respect to both low-Mg calcite and aragonite in temperatures from 25 to 64 °C. At temperatures over 42 °C, high-Mg calcite with the content of up to 15 mol% of MgCO_3_ is stable in relation to low-Mg calcite at the temperature of 60 °C (Bertram et al. 1991). The amount of MgCO_3_ also influences the solubility of high magnesium calcite. According to the results of Morse et al. (2006), the solubility of high-Mg calcite is a function of mol% of MgCO_3_ and it can exceed that of aragonite. The analysis of stability and solubility of this carbonate phase indicates that other factors than the Mg content can influence the solubility of high-Mg calcite, especially increased content of CO_2_ in ocean water (Morse et al. 2006). Low-Mg calcite and high-Mg calcite also have different crystal structure. Low-Mg calcite and high-Mg calcite have the same rhombohedral symmetry, but the unit cell parameters are different. According to Markgraf and Reeder (1985), low-Mg calcite standard cell parameters are as follows: *a*_0_ = 4.988 Ǻ, *c*_0_ = 17.061 Ǻ, *V* = 367.6(1) Ǻ^3^ and the ratio *c*_0_/*a*_0_ = 3.42. High magnesium calcite has different parameters: *a*_0_ = 4.941(2) Ǻ, *c*_0_ = 16.864(2) Ǻ, *V* = 356.60(22) Ǻ^3^ and the ratio *c*_0_/*a*_0_ = 3.413 (Althoff 1977). Because of that the peaks of low magnesium calcite and high magnesium calcite are different. According to Paquette and Reeder (1990), the amount of MgCO_3_ in biogenic high magnesium calcite fluctuates from 6.4 to 12.9 mol%—although the total amount of MgCO_3_ in high-Mg calcite could achieve 30 mol%. The results of their researches show that for 6.4 mol% content of MgCO_3_ the parameters of high-Mg calcite cell present values: *a*_0_ = 4.9673(3) Ǻ, *c*_0_ = 16.9631(7) Ǻ, *V* = 362.48(4) Ǻ^3^ and the ratio *c*_0_/*a*_0_ = 3.415. If the content of MgCO_3_ is 12.9 mol%, the parameters of high magnesium calcite crystal cell are: *a*_0_ = 4.9382(4) Ǻ, *c*_0_ = 16.832(1) Ǻ, *V* = 355.48(7) Ǻ^3^ and the ratio *c*_0_/*a*_0_ = 3.4085. According to Johnson (2002), *a*_0_ and *c*_0_ cell parameters of high-Mg calcite for 13.6% Mg substitution (47.6 mol% of MgCO_3_) are as following: *a*_0_ = 4.942 Ǻ and *c*_0_ = 16.85 Ǻ.

Low magnesium calcite is usually presented by the chemical formula—(Ca_0.998_,Mg_0.002_)CO_3_ (Markgraf and Reeder 1985). According to Althoff (1977) high-Mg calcite is presented by the chemical formula—(Ca_0.9_,Mg_0.1_)CO_3,_ but according to Morse and Mackenzie (1990) it is as follows—(Ca_0.85_,Mg_0.15_)CO_3_. It is known that high magnesium calcite and aragonite represent unstable or metastable phases. They are transformed into low magnesium calcite in normal temperature (26 °C) and pressure (to 20 atm.) (Deleuze and Brantley 1997; Fairchild et al. 2000; Zhang and Dave 2000). Moreover, aragonite and high-Mg calcite are often replaced by early diagenetic dolomite. It frequently occurs in ancient and modern environments where sulphate-evaporite deposits are strictly associated with dolomite deriving from early diagenetic processes.

To establish high-Mg calcite genesis and conditions of forming of this mineral, it is necessary to know the chemical conditions of the depositional paleoenvironment and the diagenetic processes which could generate and preserve this unstable carbonate phase, which is typical for modern sediments in Triassic deposits. The sources of magnesium are usually seawater and sometimes fresh waters. Magnesium could also come from weathered carbonate or silicate rocks. When delivered to seawater in a shelf zone, sometimes high-Mg calcite is formed (Mackenzie and Andersson 2013; Morse and Mackenzie 1990). High-Mg calcite is unstable with respect to low-Mg calcite as it may lose its Mg in time and alter to low-Mg calcite. If it is exposed to magnesium-rich pore waters, high magnesium calcite can gain additional Mg and be replaced by dolomite (Boggs 2010). Dolomite formation depends on Mg/Ca ratio, temperature, CO_2_ content, reaction time and other factors (Mackenzie and Andersson 2013; Morse and Mackenzie 1990; Tucker and Wright 1990). High magnesium calcite, which includes up to 40% of MgCO_3_, (Zhang et al. 2010) is observed in many natural low-temperature environments (Böttcher et al. 1997; Fairbridge 1957). However, these high values are mostly referred to inorganic induced carbonates. Magnesium carbonates have also formed the skeletons of a wide variety of marine organisms (Stanley 2008). Namely the skeletons of crinoids, as well as of the entire phylum of Echinodermata, are composed of high-Mg calcite skeletons (Gorzelak et al. 2013, 2016). The ossicles are made up of porous calcite plates. However, placed inside the fossil they usually become closed by diagenetic calcite in optical continuity and each plate is a monocrystal with organic macromolecules in the crystal lattice.

High-Mg calcite is an unstable mineral. Substitution of Mg^2+^ into calcite lattice has significant influence on reactivity of carbonate solids because thermodynamic stability and dissolution/precipitation kinetics are changed (Böttcher et al. 1997). For that reason, high-Mg calcite turns into low magnesium calcite during diagenetic processes and high magnesium calcite is rare in ancient carbonates; however, it is possible to be found in sediments formed under high salinity conditions. In fresh water, low in Mg, transformation of unstable minerals into stable ones does not proceed very quickly. It depends on the Mg/Ca ratio, but particularly on the solid–liquid ratio (in other words: on the flow through behavior based on permeability and porosity). In water rich in magnesium, unstable phases of carbonate minerals, like high-Mg calcite, could be preserved. Sometimes neomorphic processes could influence the preservation of high magnesium calcite. In vadose zone less-stable aragonite and low-Mg calcite are destroyed and stable high-Mg calcite is generated (Boggs 2010).

Occurrence of high magnesium calcite in the Muschelkalk limestone of the Polish part of the Germanic Basin (Opole) can indicate the special conditions of the marine water in the basin and sedimentary environment where these limestones were created (Stanienda 2006, 2011). Because the theory about the presence of the high-Mg calcite in the Muschelkalk limestone was doubtful, it was necessary to identify this mineral phase using different analytical methods.

The aim of this article is to assess the origin of high magnesium calcite (high-Mg calcite/magnesium calcite) in Muschelkalk limestones of the Polish part of the Germanic Basin with the aid of geochemical and mineralogical analyses.

### Geologic setting and sampling

**Fig. 1:**
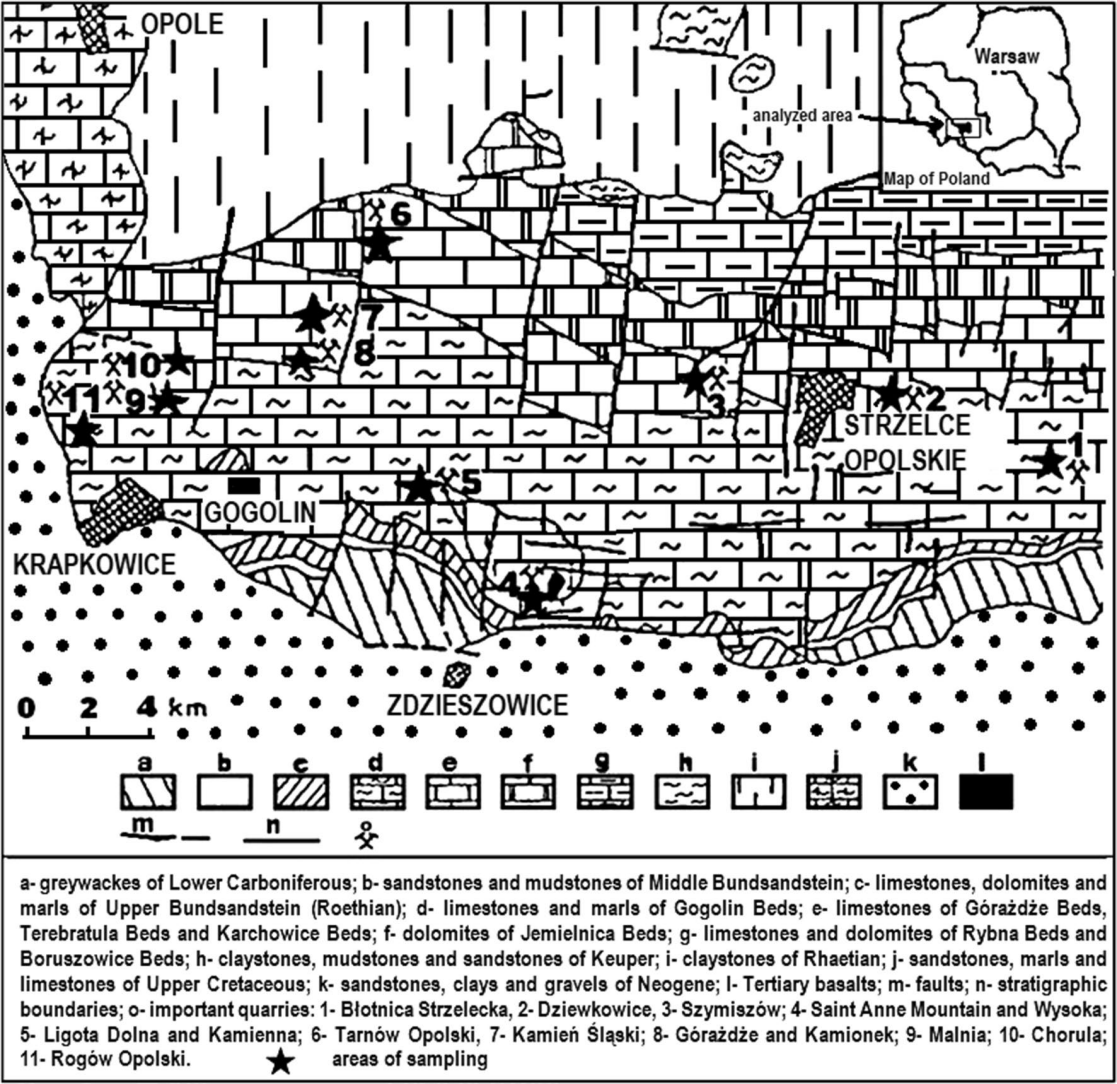
Geological map of the central part of Opole Silesia (After Niedźwiedzki 2000; Stanienda 2013; modified)

**Fig. 2:**
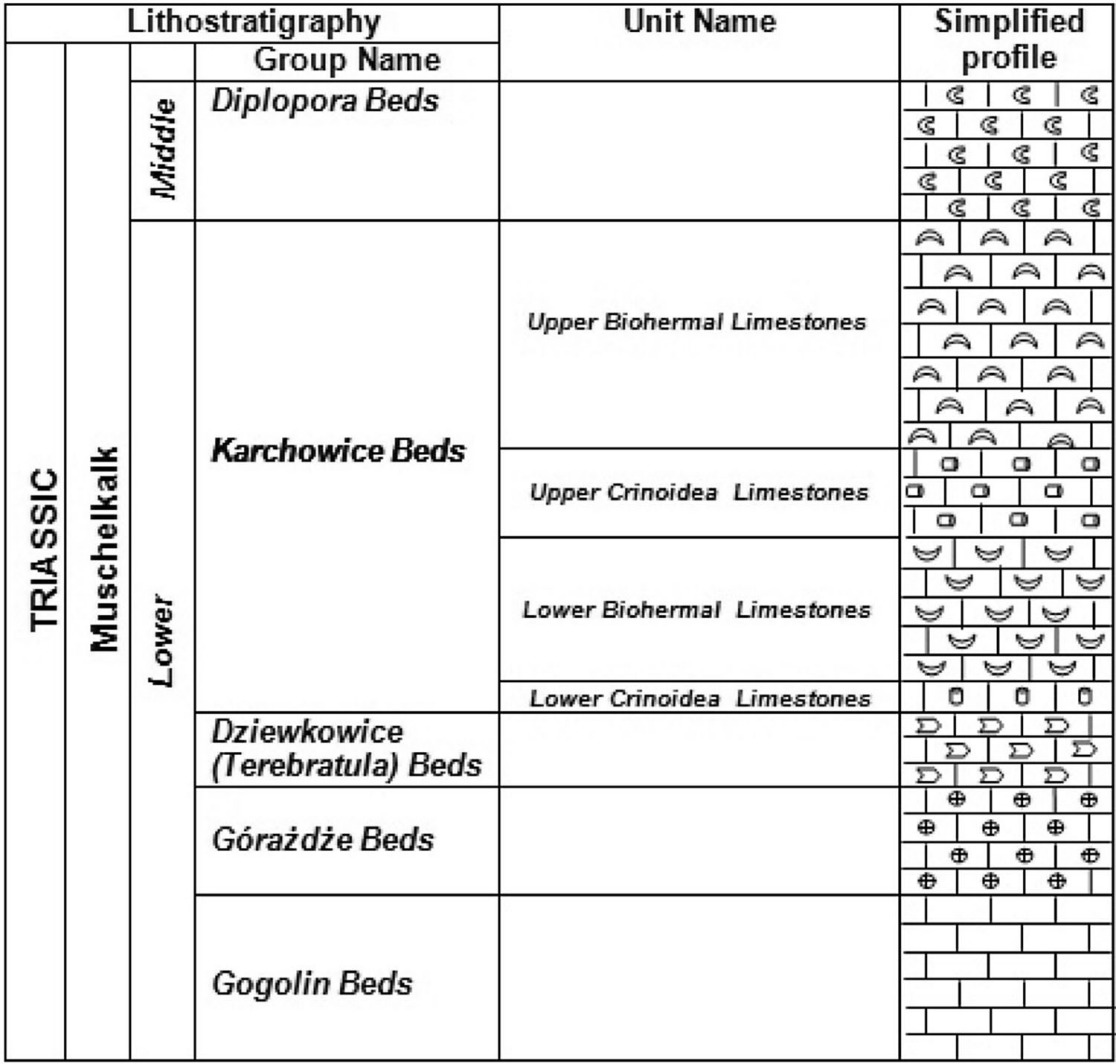
Lithostratigraphic profile of Muschelkalk sediments of the analyzed area.  Diplopora Beds;  Karchowice Beds (upper Biohermal limestones);  Karchowice Beds (upper Crinoidea limestones);  Karchowice Beds (lower Biohermal limestones);  Karchowice Beds (lower Crinoidea limestones);  Dziewkowice (Terebratula) Beds;  Górażdże Beds;  Gogolin Beds

**Fig. 3:**
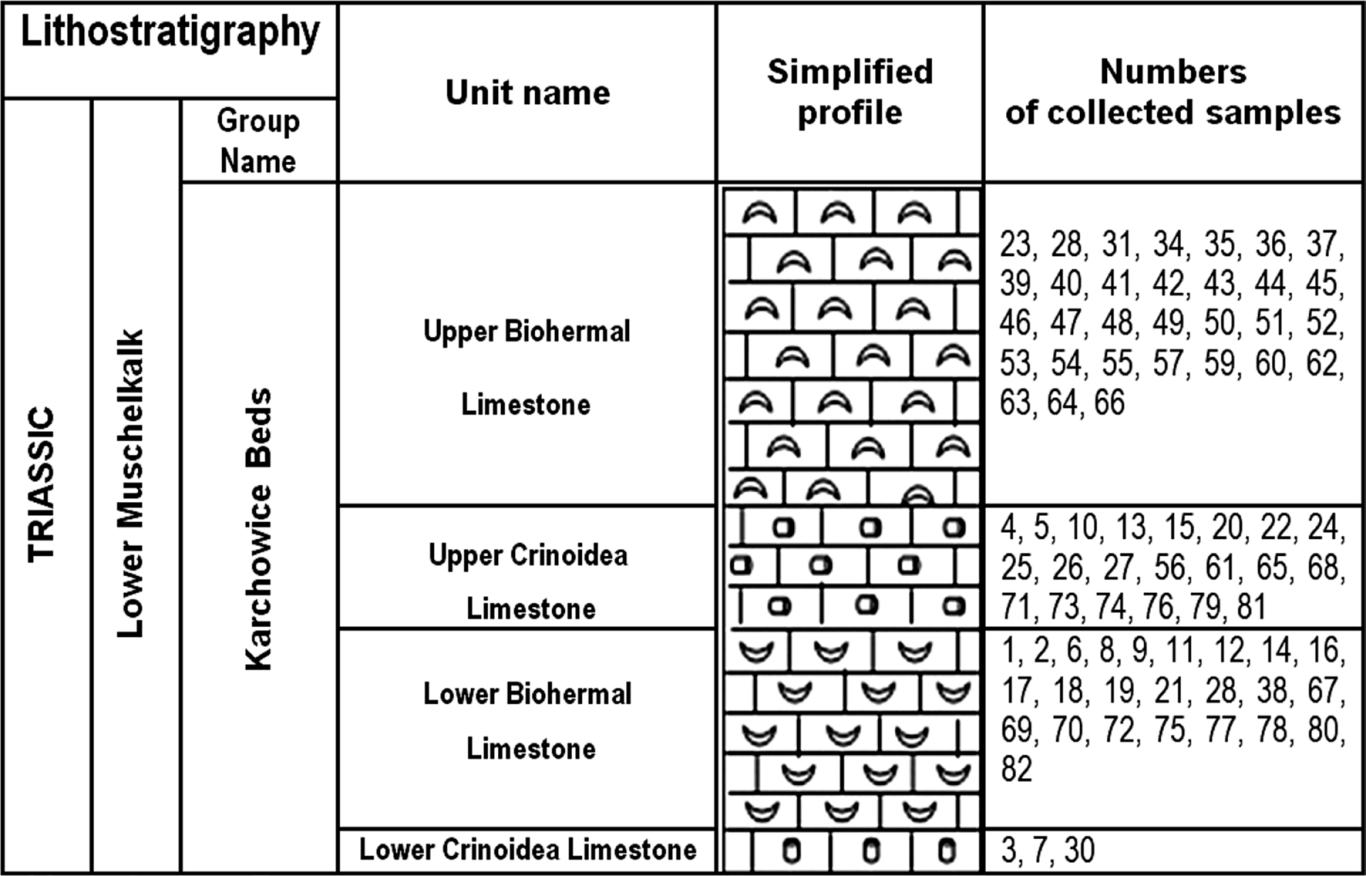
Lithostratigraphic profile of the Karchowice Beds form the Tarnów Opolski Deposit.  Karchowice Beds (upper Biohermal limestones);  Karchowice Beds (upper Crinoidea limestones);  Karchowice Beds (lower Biohermal limestones);  Karchowice Beds (lower Crinoidea limestones)

Triassic of Opole Silesia belongs to the Eastern part of the epicontinental Germanic Basin (Szulc 1990, 1993, 2000; Niedźwiedzki 2000; Feist-Burkhardt et al. 2008) (Fig. 1). The section of the Opole Silesia Triassic rocks includes Muschelkalk sediments. In this section, four stratigraphic units were distinguished: in the bottom Gogolin Beds, next-Górażdże Beds, Dziewkowice (Terebratula) Beds and Karchowice Beds (the top of section) (Figs. 2, 3). The samples for investigation were taken from the “Tarnów Opolski Deposit”, which are situated in Silesia, in the area of Opole (the south-western Poland) (Fig. 1). 82 samples were taken from “Tarnów Opolski Deposit”: 2—from Terebratula Beds, 79—from Karchowice Beds and 1—from Diplopora Beds. 79 samples collected from the Karchowice Formation were analyzed (Fig. 3). The samples from this stratigraphic unit were chosen for study because of the highest content of Mg in the rocks. Four types of beds were distinguished in this formation: Lower Crinoidea Beds, Lower Biohermal Beds, Upper Crinoidea Beds and Upper Biohermal Beds (Figs. 2, 3). Lower Crinoidea Beds are composed of beige echinoderm-bearing limestones. Lower Biohermal Beds are built of large beige or grey sponge-crinoid-coral bioherms with inter-bioherm calcirudites. Upper Crinoidea Beds include very thick-bedded, beige, shell-echinoderm limestones, partially dolomitized with large-scale cross stratification, partially dolomitized and Upper Biohermal Beds are composed mainly of beige coral bioherms (Bodzioch 2005). Each type of beds was sampled.

### Identification of high-Mg calcite

#### Methodology of the study

3 samples were taken from Lower Crinoidea Beds (3, 7 and 30), 24—from Lower Biohermal Beds (samples 1, 2, 6, 8, 9, 11, 12, 14, 16, 17, 18, 19, 21, 28, 38, 67, 69, 70, 72, 75, 77, 78, 80, 82), 21—from Upper Crinoidea Beds (samples 4, 5, 10, 13, 15, 20, 22, 24, 25, 26, 27, 56, 61, 65, 68, 71, 73, 74, 76, 79, 81) and 31—from Upper Biohermal Beds (samples 23, 28, 31, 34, 35, 36, 37, 39, 40, 41, 42, 43, 44, 45, 46, 47, 48, 49, 50, 51, 52, 53, 54, 55, 57, 59, 60, 62, 63, 64, 66) (Fig. 3). Samples weighing 1–2 kg were taken.

Measurements were carried out by means of microscopic analysis, FTIR, X-ray analyses, microprobe measurements including mapping based on microprobe measurements and ICP spectrometry.

The microscopic analysis was executed using an Axioskop Universal Microscope produced by Zeiss equipped with a photo camera. All samples were studied using the microscope. FTIR was done using Fourier Transform Spectrometer Bio-Rad FTS 135 which had 2 cm^−1^ resolution with a typical range of 400–4000 cm^−1^. Six samples were investigated by means of this method: two of lower Crinoidea limestones (samples 7 and 30), one of lower Biohermal limestones (sample 1), one of upper Crinoidea limestones (sample 22) and two samples of upper Biohermal limestones (samples 41 and 62) (Stanienda 2005). X-ray diffraction was carried out using an X-ray diffractometer XRD 7 made by Seifert-FPM. Measurements were executed with a range of *d*-spacing (Å) from 0.8563 to 0.0953 nm. Using this equipment it was possible to identify mineral phases but without an opportunity to determine the crystal structure. Four samples were investigated by means of this method: one of lower Crinoidea limestones (sample 7), two samples of upper Crinoidea limestones (samples 15 and 22) and one of upper Biohermal limestones (sample 62) (Stanienda 2006). Microprobe measurements were done using JEOL-JCXA-733 microprobe equipped with a Link ISIS 300 spectrometer made by Oxford Instruments. The investigations were carried out on polished sections which were sputtered with a carbon coat. Four samples were investigated by this method: lower Crinoidea limestone (sample 7), lower Biohermal limestone (sample 1), upper Crinoidea limestone (sample 15) and upper Biohermal limestone (sample 41). The WDS method was applied to carry out quantitative analyses in micro areas with the selected points having different chemical composition. The content of the following chemical elements was determined: O, C, Mg, Si, Ca, Fe, and for some samples also K, Ba, Sr, Mn, Al, Na, Cu, Zn, Pb, Cd and Y. Mapping was executed using microprobe measurements method. The analysis was carried out applying the techniques of X-ray microanalysis EPMA, with the application of an X-ray microanalyzer JXA-8230 manufactured by JOEL. The analyses were carried out on polished sections which were sputtered with a carbon coat. Two samples were investigated with this method: lower Crinoidea limestone (sample 7) and upper Biohermal limestone (sample 41). The EDS analysis was carried out. The X-ray maps of one investigated microarea of sample 1 and two microareas of sample 5 were made using the EDS spectrometer. The content of the following elements was determined: O, C, Mg, Ca and Fe. ICP spectrometry was carried out using an emission spectrometer ICP-AES of JY 2000 type with excitation in plasma induced conjugate. The measurement was based on atomic emission. Four samples were investigated by means of this method: lower Crinoidea limestone (sample 7), upper Crinoidea limestone (sample 22), lower Biohermal limestone (sample 1) and upper Biohermal limestone (sample 41). The content of the following elements was determined: Ca, Mg, Fe, Mn, Sr and Ba.

### Results of the study

#### Microscopic analysis

**Fig. 4:**
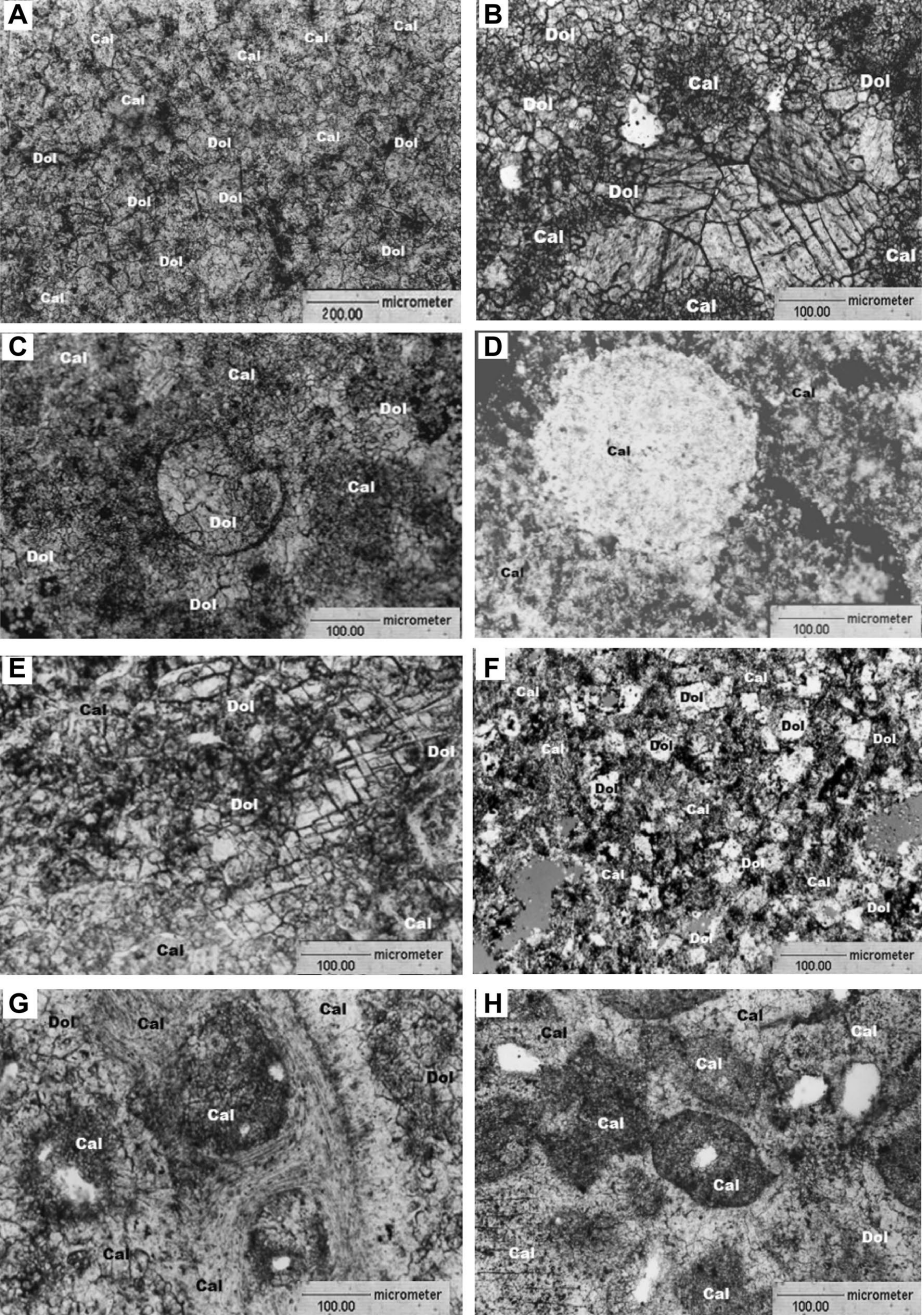
**A** Microscopic view of lower Crinoidea limestone (sample 7)—limestone built of sparry mass, built mainly of calcite crystals and rhombohedral dolomite crystals. 1N, Magn. ×200 (Stanienda 2000, 2011). Cal, calcite phase; Dol, dolomite phase. **B** Microscopic view of lower Biohermal limestone (sample 2)—aggregates of coarse-grained calcite crystals and rhombohedral dolomite crystals in sparry rock mass. XN, Magn. ×100 (Stanienda 2000, 2011). Cal, calcite phase; Dol, dolomite phase. **C** Microscopic view of lower Biohermal limestone (sample 8)—bioclast filled with coarse-grained calcite, in some places also dolomite in microsparry orthochem cement built mainly of calcite crystals and in some areas of rhombohedral dolomite crystals. XN, Magn. ×100 (Stanienda 2000, 2011). Cal, calcite phase; Dol, dolomite phase. **D** Microscopic view of upper Crinoidea limestone (sample 15)—in the foreground bioclast (crinoid circular stem plate) in microsparry orthochem cement built of calcite. XN, Magn. ×100 (Stanienda 2000, 2011). Cal, calcite phase. **E** Microscopic view of upper Crinoidea limestone (sample 61)—on the right side vein filled with sparry calcite with visible rhombohedral cleavage. Apart from calcite crystals also rhombohedral dolomite crystals are visible in some places. XN, Magn. ×100 (Stanienda 2000, 2011). Cal, calcite phase; Dol, dolomite phase. **F** Microscopic view of upper Biohermal limestone (sample 41)—rhombohedral dolomite crystals dominate here. In some areas micro-sparry and micritic calcite is visible. XN, Magn. ×100 (Stanienda 2000, 2011). Cal, calcite phase; Dol, dolomite phase. **G** Microscopic view of upper Biohermal limestone (sample 57)—bioclast in sparry rock mass. In some places rhombohedral dolomite crystals are visible. XN, Magn. ×100 (Stanienda 2000, 2011). Cal, calcite phase; Dol, dolomite phase. **H** Microscopic view of upper Biohermal limestone (sample 64)—group of bioclasts cemented by sparry, in some areas of rock, syntaxial calcite. In some places rhombohedral dolomite crystals are visible. XN, Magn. ×100 (Stanienda 2000, 2011). Cal, calcite phase; Dol, dolomite phase

Results of the microscopic analysis (Fig. 4) allowed to distinguish only two carbonate phases: calcite and dolomite. The calcite phase includes low magnesium calcite and Mg-calcite. Micritic grains of calcite usually form rock mass in limestones poor in allochems (Fig. 4A, B, E and F) and cement which forms bioclasts (Fig. 4C, D, G and H) in limestones with allochems. Bigger, often sparry probably low magnesium calcite grains form veins or aggregates. They are usually different in shape and size (Fig. 4B, E). It is probably secondary calcite, a product of diagenetic processes, aggradation and recrystallization of primary micritic crystals. In some calcite crystals a rhombohedral cleavage is visible. Calcite crystals sometimes form palisade cement which surrounds allochems. Fragments of crinoids: columnals (Fig. 4D, H) or crown plates (Fig. 4C, G) usually dominate among bioclasts. Allochems are often filled with micrite. According to Dunham (1962) and Embry and Klovan (1971) classifications the analyzed limestones of Lower and Upper Crinoidea Beds represent crinoidal packstones. In Flügel’s classification, the rocks represent standard microfacies of SMF 12 type (limestones with shell concentrations). Most echinoderm accumulations are composed of crinoids (SMF 12-CRIN) (Fig. 4D). According to Dunham (1962) and Embry and Klovan (1971) classifications limestones of Lower and Upper Biohermal Beds also represent packstones. In Flügel’s classification the rocks represent standard microfacies type SMF 12 (limestones with shell concentrations). They belong to the SMF12-S group (coquina composed of shells without specific assignment) (Flügel 2004) (Fig. 4C, G and H). Rocks of analyzed facies could be formed in various settings, including slopes, protected platforms, reefs and mounds and therefore also in the shallow epicontinental basin. This type of limestone can indicate in-place degradation or deposition subsequent to transport (Flügel 2004).

Microscopic analysis is also allowed to identify dolomite. All sparry rhombohedral crystals are the dolomite phase. These crystals are usually well visible in thin sections (Fig. 4A, B). In some samples it is possible to observe calcite pseudomorphs after dolomite (dedolomites). Some of them are filled with iron oxides.

The results of the microscopic study show that calcite phases dominate in most of the investigated limestone. The higher content of dolomite was observed in lower Crinoidea limestone and lower Biohermal limestone. In some studied samples also smaller amounts of quartz and clay minerals were observed.

#### Fourier transform spectroscopy

Results of FTIR indicate that high magnesium calcite is present in limestones of all investigated strata. It was identified in five samples (Table 1) (Stanienda 2005). High magnesium calcite is present in smaller amounts that low magnesium calcite. In lots of samples dolomite was also identified (Table 1).

**Table 1:** Results of the Fourier transform infrared spectroscopy (FTIR) (cm^−1^) (Stanienda 2005)

Type of limestone	Low-Mg calcite	High-Mg calcite	Dolomite	Quartz	Clay minerals
Lower Crinoidea limestone (sample 7—content of MgO: 9.5%)	*V*_4_ = 712.39 *V*_2_ = 848 *V*_2_ = 876.12 *V*_1_ + *V*_4_ = 1798.41 Other absorption bands—2599, 2875.66; 2928.29; 2983.72; 3422.71	*V*_3_ = 1428.23 *V*_1_ + *V*_3_ = 2517.35	*V*_4_ = 728.77 *V*_1_ + *V*_4_ = 1822.95	465.86 779.33 799.59 1095.22	570.08 577.27 604.41 923.04 965.14 1047.34
Lower Biohermal limestone (sample 1—content of MgO: 7.36%)	*V*_4_ = 712.39 *V*_2_ = 847.94 *V*_2_ = 876.19 *V*_1_ + *V*_4_ = 1798.23 *V*_1_ + *V*_3_ = 2512.36 Other absorption bands—2587.82; 2875.01; 2946.62; 2983.26 3418.49	*V*_3_ = 1428.34	–	463.28 515.05 777.40 798.69 1094.38	570.49 604.58 965.62 1049.06 1169.97
Upper Crinoidea limestone (sample 22—content of MgO: 11.34%)	*V*_4_ = 712.55 *V*_2_ = 848.37 *V*_2_ = 876.96 *V*_1_ + *V*_4_ = 1797.56 Other absorption bands—2519.87; 2577.21; 2876.87; 2984.23; 3448.5	*V*_3_ = 1434.81 *V*_1_ + *V*_3_ = 2519.87 Other absorption bands—2621.57; 2942.76	*V*_4_ = 728.77 *V*_2_ = 852.98 *V*_2_ = 880 *V*_1_ + *V*_4_ = 1818.13	1094.44	570.45 604.99 1050.18
Upper Biohermal limestone (sample 62—content of MgO: 13.95%)	*V*_4_ = 712.58 *V*_2_ = 848.18 *V*_2_ = 877.89 *V*_1_ + *V*_4_ = 1796.83 Other absorption bands—2583.96; 2877.64; 2932.15; 2984.23; 3446.71	*V*_3_ = 1435.33 *V*_1_ + *V*_3_ = 2521.2 Other absorption bands—2625.43; 3021.85	*V*_4_ = 728.86 *V*_2_ = 853 *V*_1_ + *V*_4_ = 1821.98	1094.24	569.06 605.18 1049.89

The values of the *V*_3_ absorption band of high-Mg calcite are as follows: 1433 and 1435 cm^−1^ for upper Biohermal limestone and 1428 cm^−1^ for limestones of other formations (lower Crinoidea limestone, upper Crinoidea limestone and lower Biohermal limestone) (Stanienda 2005).

The change of the *V*_3_ value can indicate the variable content of magnesium in the crystals of high magnesium calcite. The increase in the *V*_3_ value indicates the increase in the amount of Mg ions in the Mg-calcite crystal structure. The values of the *V*_3_ absorption band show a higher content of Mg in high magnesium calcite of upper Biohermal limestone in comparison with this carbonate phase of other limestone formations. The values of the *V*_3_ absorption band of high-Mg-calcite are as follows-1433 and 1435 cm^−1^ for upper Biohermal limestone and 1428 cm^−1^ for limestones of other formations (lower Crinoidea limestone, upper Crinoidea limestone and lower Biohermal limestone) (Stanienda 2005).

High-Mg calcite was also identified by the absorption band *V*_1_ + *V*_3_, which presented values from 2517 to 2521 cm^−1^ (Table 1). In some samples there also occurred other absorption bands, typical for high-Mg calcite (Table 1). Apart from carbonate phases also quartz and clay minerals were identified. The results of FTIR confirmed the hypothesis of the presence of high-Mg calcite in studied limestones.

#### X-ray diffraction

X-ray diffraction is usually used to determine crystal structure. I can refine the structure using Rietveld method, for example, or refine the lattice parameters, using the Le Bail method. The *d*_104_ value of high-Mg calcite depends on MgCO_3_ content. According to Zhang et al. (2010), a value of *d*_104_—2.988 Å is typical for high-Mg calcite with content of MgCO_3_: 16.3 mol%, a value of *d*_104_—2.972 Å for content of MgCO_3_: 22.5 mol%. If the content of MgCO_3_ rises, the value of *d*_104_ drops. Unstable calcite phases could also present the value of *d*_104_—2.964 Å (Smyth and Ahrens 1997).

The results of the X-ray diffraction indicate that the peaks of high magnesium calcite show lower values than the peaks of low-Mg calcite but higher than the ones typical for dolomite (Table 2). High-Mg calcite was identified mainly based on the peaks typical for this carbonate phase (Table 2) (Stanienda 2006). The significant difference in the content of magnesium in high-Mg calcite of limestones from Karchowice Formation was not observed because the peak value of *d*_104_ appearing in X-ray diffraction patterns of investigated limestones is in all samples the same: 2.990 Å (100). With the increase of magnesium in high-Mg calcite, the peak value of *d*_104_ drops. It was impossible to calculate definitely the cell parameters of high magnesium calcite since polymineral samples were analyzed. There was a small amount of high-Mg calcite in the investigated limestones. Low-Mg calcite and high-Mg calcite were mixed in rocks, so it was not possible to separate a single crystal from this mixture. On the basis of the peak values, mainly of *d*_104_, visible in X-ray diffraction patterns of the investigated limestones, *a*_0_ and *c*_0_ values were established according to ICDD card index (ICDD card index from the PDF-4+ database with number 04-008-806). These values are as follows: *a*_0_ = 4941 Å, *c*_0_ = 16,854 Å. These values are similar to the parameters established by Johnson (2002) (*a*_0_ = 4.942 Å and *c*_0_ = 16.85 Å).

**Table 2:** Results of X-ray diffractometry

Type of sample	Type of mineral	*d*_hkl_-index of the lattice intervals of exposed parallel lattice planes system-Ǻ (*I*)-intensity of diffraction lines
Lower Crinoidea limestone (sample 7)	Low-Mg calcite	3.865 (12), 3.042 (100), 2.855 (3), 2.495 (12), 2.287 (15), 2.095 (15), 1.928 (5), 1.914 (15), 1.876 (15), 1.627 (4), 1.603 (8), 1.525 (5), 1.513 (4), 1.473 (2), 1.440 (5), 1.338 (2), 1.235 (1), 1.180 (3), 1.154 (3), 1.142 (1), 1.062 (1), 1.046 (3), 1.035 (3)
	High-Mg calcite	2.990 (100), 2.570 (2), 2.260 (15), 1.857 (15), 1.471 (2), 1.423 (3), 1.410 (3), 1.249 (1), 1.193 (3), 1.130 (1), 1.013 (2)
	Dolomite	2.895 (100), 2.684 (4), 2.544 (3), 2.410 (7), 2.195 (15), 2.069 (3), 2.027 (10), 1.807 (10), 1.789 (12), 1.470 (2), 1.433 (1), 1.392 (2), 1.296 (2), 1.273 (2), 1.202 (3), 1.125 (1), 1.111 (1), 1.009 (4), 1.0014 (5), 0.9766 (3), 0.9664 (5), 0.9642 (1)
Upper Crinoidea limestone (sample 15)	Low-Mg calcite	3.870 (12), 3.041 (100), 2.837 (3), 2.496 (12), 2.288 (15), 2.096 (15), 1.925 (5), 1.912 (15), 1.876 (15), 1.626 (4), 1.604 (8), 1.522 (5), 1.513 (4), 1. 473 (2), 1.440 (5), 1.234 (1), 1.180 (3), 1.154 (3), 1.143 (1), 1.046 (3), 1.035 (3), 0.9839 (1)
	High-Mg calcite	2.990 (100), 2.570 (2), 1.857 (15), 1.471 (2), 1.422 (3), 1.410 (3), 1.247 (1), 1.190 (3), 1.130 (1), 1.063 (1), 1.042 (1), 1.013 (2)
	Dolomite	2.893 (100), 2.020 (10), 1.470 (2), 1.340 (1), 1.298 (2), 1.099 (1), 0.9756 (3), 0.9645 (5)
	Quartz	3.355 (100)
Upper Crinoidea limestone (sample 22)	Low-Mg calcite	3.868 (12), 3.039 (100), 2.853 (3), 2.497 (12), 2.286 (15), 2.096 (15), 1.929 (5), 1.914 (15), 1.877 (15), 1.626 (4), 1.604 (8), 1.521 (5), 1.517 (4), 1.441 (5), 1.335 (2), 1.178 (3), 1.153 (3), 1.048 (3)
	High-Mg calcite	2.990 (100), 2.576 (2), 2.260 (15), 1.470 (2), 1.423 (3), 1.249 (1), 1.190 (3), 1.160 (3), 1.130 (1), 1.010 (2)
	Dolomite	4.040 (1), 3.715 (4), 3.681 (4), 2.899 (100), 2.680 (4), 2.544 (3), 2.410 (7), 2.198 (15), 2.069 (3), 2.018 (10), 1.800 (10), 1.790 (12), 1.570 (2), 1.546 (4), 1.467 (2), 1.414 (3), 1.388 (15), 1.297 (2), 1.271 (2), 1.240 (5), 1.203 (3), 1.111 (5), 1.069 (1), 1.0098 (4), 1.0018 (5), 0.9754 (3), 0.9661 (1), 0.9547 (1)
	Quartz	3.340 (100)
Upper Biohermal limestone (sample 62)	Low-Mg calcite	3.862 (12), 3.044 (100), 2.495 (12), 2.285 (15), 2.095 (15), 1.927 (5), 1.911 (15), 1.875 (15), 1.605 (8), 1.525 (5), 1.339 (2), 1.152 (3), 1.042 (3)
	High-Mg calcite	2.990 (100), 2.570 (2), 2.260 (15), 1.854 (15), 1.470 (2), 1.420 (3), 1.410 (3), 1.190 (3), 1.130 (1), 1.013 (2)
	Dolomite	3.700 (4), 2.896 (100), 2.680 (4), 2.547 (3), 2.412 (7), 2.197 (15), 2.066 (3), 2.019 (10), 1.808 (10), 1.789 (12), 1.547 (4), 1.468 (2), 1.431 (1), 1.389 (15), 1.296 (2), 1.237 (5), 1202 (3), 1.168 (1), 1.111 (5), 1.096 (1), 1.068 (1), 1.0094 (4), 1.0015 (5), 0.9744 (3), 0.9627 (5)

Apart from carbonate phases with different magnesium contents (low-Mg calcite, high-Mg calcite and dolomite), quartz was also identified.

Results of X-ray diffraction confirmed the hypothesis of the high magnesium calcite presence in the Muschelkalk limestones of the Polish part of Germanic Basin.

#### Microprobe measurements

Results of microprobe measurements show that in samples of all formations three carbonate phases are present-low magnesium calcite, high-Mg calcite and dolomite. These phases are different in respect of MgCO_3_ content.

**Fig. 5:**
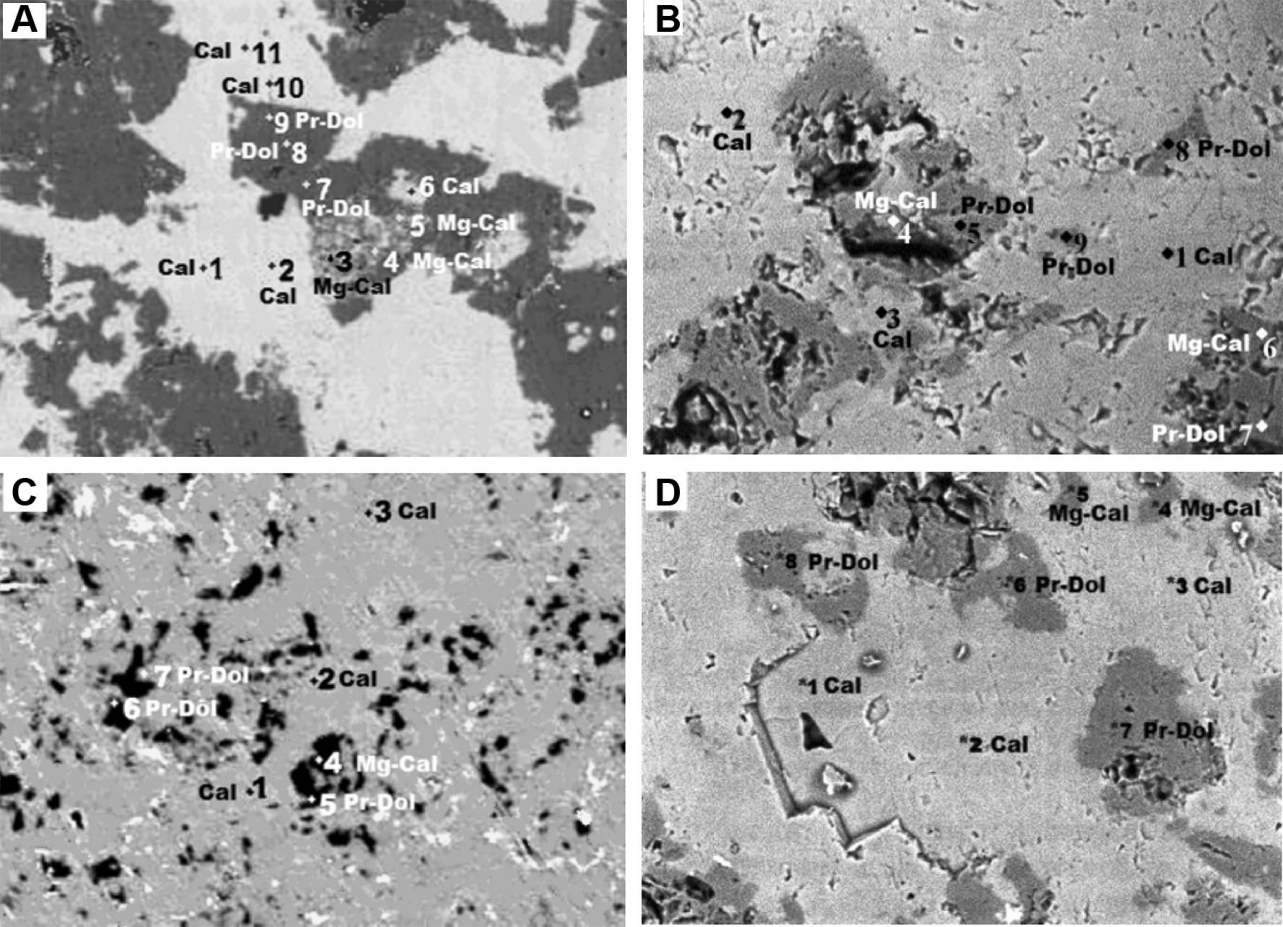
**A** BSE image of an lower Crinoidea limestone (sample 7) (Stanienda 2004, 2011). Magn. ×1500, 1–11-points of chemical analysis. Cal—low-Mg calcite; Mg-Cal—high-Mg calcite; Pr-Dol—protodolomite. **B** BSE image of an Lower Biohermal Limestone (sample 2). Magn. ×600, 1–9-points of chemical analysis. Cal—low-Mg calcite; Mg-Cal—high-Mg calcite; Pr-Dol—protodolomite. **C** BSE image of an upper Crinoidea limestone (sample 15) (Stanienda 2004, 2011). Magn. ×1000, 1–7-points of chemical analysis. Cal—low-Mg calcite; Mg-Cal—high-Mg calcite; Pr-Dol—protodolomite. **D** BSE image of an upper Biohermal limestone (sample 41). Magn. ×1500, 1–8-points of chemical analysis (Stanienda 2006). Cal—low-Mg calcite; Mg-Cal—high-Mg calcite; Pr-Dol—protodolomite

In lower Crinoidea limestone: Variability of mineral phases showing different amounts of magnesium is possible to observe in BSE image. The phases reveal different shades of grey. Fair grey characterizes low magnesium calcite. Grey is typical for high-Mg calcite and dark grey—for dolomite (Fig. 5A). It was confirmed by results of chemical analysis executed in selected points of microarea (Table 3). Micritic, xenomorphic crystals of high magnesium calcite form rock cement. Dolomite, sparry crystals are euhedral and rhombohedral in shape (Fig. 5A). In lower Crinoidea limestone, the content of MgCO_3_ in low-Mg calcite ranges from 1.02 to 8.22%, in high-Mg calcite—from 22.82 to 33.11% and in dolomite—from 44.13 to 45.78% (Table 3). The low content of MgCO_3_ in the dolomite phase in comparison with the stoichiometric value for dolomite (46.13% MgCO_3_, 13.18% Mg) indicates that probably protodolomite is present in lower Crinoidea limestone. This non-stoichiometric, poorly ordered carbonate phase usually forms crystals euhedral and rhombohedral in shape (Boggs 2010; Morse and Mackenzie 1990; Tucker and Wright 1990). The amount of Ca in high magnesium calcite ranges from 30.30 to 36.06%, Mg: from 6.52 to 9.46%, C: from 7.80 to 8.80% and O: from 48.70 to 50.41%. The median value of magnesium calculated for high-Mg calcite averages to 8.37% (29.30% MgCO_3_). In protodolomite and high-Mg calcite the increased content of iron was observed (Table 3). The iron probably replaces magnesium in these carbonate phases.

**Table 3:** Microprobe chemical analyses of the lower Crinoidea limestone (sample 7) (Stanienda 2004, 2011)

Chemical elements	Number of analyzed points according to fig. 11 [% mass.]										
	1 Low-Mg calcite	2 Low-Mg calcite	3 High-Mg calcite	4 High-Mg calcite	5 High-Mg calcite	6 Low-Mg calcite	7 Protodolomite	8 Protodolomite	9 Protodolomite	10 Low-Mg calcite	11 Low-Mg calcite
C	4.31	8.72	7.80	9.14	8.51	9.09	7.76	7.49	7.70	8.43	8.80
Mg	0.29	0.44	6.52	9.14	9.46	2.35	13.08	12.91	12.61	0.35	0.32
Si	0.09	0.07	0.15	0.71	0.24	0.13	0.08	0.08	0.12	0.04	0.07
Ca	42.36	41.06	36.06	30.48	30.30	39.54	25.96	25.36	25.81	40.81	40.06
Mn	0.00	0.03	0.01	0.03	0.05	0.09	0.00	0.01	0.13	0.02	0.01
Fe	0.14	0.01	0.61	0.72	0.82	1.14	0.50	0.80	0.76	0.13	0.03
Cu	0.11	0.08	0.03	0.03	0.03	0.17	0.09	0.11	0.12	0.12	0.18
Zn	0.08	0.05	0.01	0.13	0.00	0.16	0.04	0.20	0.06	0.06	0.19
Y	0.13	0.02	0.05	0.07	0.02	0.08	0.08	0.19	0.02	0.07	0.05
Cd	0.06	0.05	0.06	0.03	0.09	0.02	0.02	0.02	0.02	0.25	0.23
Pb	0.04	0.02	0.00	0.01	0.07	0.03	0.08	0.00	0.01	0.05	0.05
O	52.39	49.45	48.70	49.51	50.41	47.20	52.31	52.83	52.64	49.67	50.01
Total	100.00	100.00	100.00	100.00	100.00	100.00	100.00	100.00	100.00	100.00	100.00
MgCO_3_ content	1.02	1.54	22.82	31.99	33.11	8.22	45.78	45.18	44.13	1.22	1.12

The results of the analyses allowed to determine a high magnesium calcite chemical formula of lower Crinoidea limestone: (Ca_0.87–0.80_,Mg_0.13–0.20_)CO_3_.

In lower Biohermal limestone: low-Mg calcite and high-Mg calcite form the micritic rock mass. The mixture of these carbonate phases is visible in BSE image as a fair grey mass (Fig. 5B), but there is no difference in colour shades between low-Mg calcite and high-Mg calcite. Sparry dark grey dolomite crystals (Table 4) are euhedral and rhombohedral in shape (Fig. 5B). Like in lower Crinoidea limestone, also in these rocks protodolomite is probably present. The crystals of this carbonate phase are similar to protodolomite crystals of lower Crinoidea limestone. The difference in the magnesium content in carbonate phases is only possible to observe in Table 4 which includes the results of the chemical analysis executed in selected points of the microarea. In lower Biohermal limestone the content of MgCO_3_ in low magnesium calcite ranges from 0.42 to 1.19%, in high-Mg calcite—from 29.61 to 36.89% (Table 4). The amount of Ca in high magnesium calcite ranges from 33.46 to 36.18%, Mg: from 8.46 to 10.54%, C: from 7.95 to 10.28% and O: from 43.51 to 46.86%. The median value of magnesium calculated for high-Mg calcite averages 9.5% (33.25% MgCO_3_). Protodolomite was also identified in this limestone. It is represented by crystals in which the content of MgCO_3_ ranges from 40.60 to 43.92% (Table 4). The content of MgCO_3_ in protodolomite is lower than the stoichiometric value for dolomite—46.13%.

**Table 4:** Microprobe chemical analyses of the lower Biohermal limestone (sample 1)

Chemical elements	Numbers of analyzed points according to fig. 12 [% mass.]								
	1 Low-Mg calcite	2 Low-Mg calcite	3 Low-Mg calcite	4 High-Mg calcite	5 Protodolomite	6 High-Mg calcite	7 Protodolomite	8 Protodolomite	9 Protodolomite
C	11.09	13.10	12.77	10.28	12.03	7.95	10.95	11.83	12.91
Mg	0.34	0.12	0.29	8.46	11.70	10.54	11.98	12.55	11.60
Si	0,06	0.14	0.09	0.22	0.24	0.25	0.22	1.46	0.02
Ca	40.06	36.84	37.68	33.46	24.93	36.18	27.54	21.91	21.90
Fe	0.11	0.02	0.12	0.72	0.78	1.57	0.71	3.05	0.98
O	48.34	49.78	49.05	46.86	50.32	43.51	48.60	49.20	52.59
Total	100.00	100.00	100.00	100.00	100.00	100.00	100.00	100.00	100.00
MgCO_3_ content	1.19	0.42	1.01	29.61	40.95	36.89	41.93	43.92	40.60

As in the previous sample, also in this case the increased content of iron was observed in high-Mg calcite and protodolomite (Table 4). The iron probably replaces magnesium in these carbonate phases.

The results of these analyses allowed to determine a high magnesium calcite chemical formula of lower Biohermal limestone: (Ca_0.83–0.75_,Mg_0.17–0.25_)CO_3_.

Upper Crinoidea limestone: Is also composed of three carbonate phases. Low-Mg calcite and high-Mg calcite form the micritic rock mass, similar to the rock mass of lower Biohermal limestone. In BSE image, it is not possible to distinguish these carbonate phases based on the shades of grey colour (Fig. 5C), similarly to the previous sample. Small sparry dolomite crystals, almost black in colour in this sample, are euhedral and rhombohedral in shape (Fig. 5C). The difference in the magnesium content in the carbonate phases may be seen in Table 5, which presents the results of chemical analysis executed in the selected points of the microarea. In upper Crinoidea limestone the content of MgCO_3_ in low magnesium calcite ranges from 1.43 to 1.85%, in high-Mg calcite—it is 30.52% and in dolomite phase—from 43.33 to 45.57% (Table 5). In upper Crinoidea limestones, protodolomite is also present. High magnesium calcite of upper Crinoidea limestone is composed of 29.30% of Ca, 8.72% of Mg, 10.08% of C and 50.09% of O. Sodium occurred in the upper Crinoidea limestone may come from diagenetic fluids. The occurrence of Ba and Sr in rocks confirms the presence of the aragonite phase in the primary carbonate sediment. The results allowed to determine a high magnesium calcite chemical formula of upper Crinoidea limestone: (Ca_0.81_,Mg_0.19_)CO_3_.

**Table 5:** Microprobe chemical analyses of the upper Crinoidea limestone (sample 15) (Stanienda 2004, 2011)

Chemical elements	Number of analyzed points according to Fig. 13 [% mass.]						
	1 Low-Mg calcite	2 Low-Mg calcite	3 Low-Mg calcite	4 High-Mg calcite	5 Protodolomite	6 Protodolomite	7 Protodolomite
C	10.04	10.02	9.99	10.08	6.11	6.48	8.13
Na	0.98	0.93	0.95	1.24	0.93	1.02	1.19
Mg	0.41	0.53	0.45	8.72	12.38	13.02	12.68
Al	0.05	0.05	0.05	0.09	0.07	0.06	0.05
Si	0.07	0.07	0.06	0.12	0.09	0.06	0.08
Ca	41.16	40.86	41.17	29.30	27.69	27.29	27.63
Mn	0.04	0.09	0.04	0.01	0.03	0.05	0.16
Fe	0.02	0.21	0.21	0.27	0.37	0.36	0.18
Sr	0.05	0.00	0.03	0.02	0.13	0.02	0.05
Ba	0.01	0.02	0.10	0.06	0.17	0.09	0.07
O	47.17	47.22	46.95	50.09	52.03	51.55	49.78
Total	100.00	100.00	100.00	100.00	100.00	100.00	100.00
MgCO_3_ content	1.43	1.85	1.57	30.52	43.33	45.57	44.38

Three carbonate phases, varying in amount of MgCO_3_, were also identified in upper Biohermal limestone. Low-Mg calcite and high-Mg calcite form, the micritic rock mass. In BSE image of this limestone there is no difference in the colour shades of calcite phases (Fig. 5D). They form fair grey mass. Dolomite phase is easy to distinguish in BSE image. It forms dark grey, sparry crystals which are euhedral and rhombohedral in shape (Fig. 5D). Looking at the results of the chemical analysis (Table 6), one can observe the difference in magnesium content in the carbonate phases, as well as in this limestone. In upper Biohermal limestone the content of MgCO_3_ in low-Mg calcite ranges from 0.14 to 0.21%, in high-Mg calcite—from 30.76 to 34.02% and in dolomite phase—from 42.42 to 43.68% (Table 6). However, the dolomite phase is characterized by a lower content of MgCO_3_ in comparison with the stoichiometric value for dolomite, so this mineral should be treated as protodolomite. The amount of Ca in high magnesium calcite of upper Crinoidea limestone ranges from 26.10 to 26.18%, Mg: from 8.79 to 9.72%, C: from 12.83 to 13.00% and O: from 51.11 to 51.21%. The median value of magnesium calculated for high-Mg calcite averages to 9.25% (32.37% MgCO_3_). Iron was not identified in the analyzed limestone sample with low-Mg calcite. Small amounts of this element occur in protodolomite and high-Mg calcite (Table 6). The iron probably replaces magnesium in these carbonate phases.

**Table 6:** Microprobe chemical analyses of the upper Biohermal limestone (sample 41) (Stanienda 2006)

Chemical elements	Number of analyzed points according to Fig. 14 [% mass.]							
	1 Low-Mg calcite	2 Low-Mg calcite	3 Low-Mg calcite	4 High-Mg calcite	5 High-Mg calcite	6 Protodolomite	7 Protodolomite	8 Protodolomite
C	11.16	12.19	12.21	13.00	12.83	12.94	12.88	13.12
Mg	0.04	0.06	0.05	8.79	9.72	12.48	12.22	12.12
Si	0.00	0.00	0.00	0.00	0.00	0.00	0.00	0.00
Ca	40.19	39.40	39.34	26,18	26.10	22.70	23.05	22.48
Fe	0.00	0.00	0.00	0.81	0.24	0.10	0.21	0.27
Y	0.01	0.08	0.09	0.01	0.00	0.00	0.00	0.00
O	48.60	48.27	48.31	51.21	51.11	51.78	51.64	52.01
Total	100.00	100.00	100.00	100.00	100.00	100.00	100.00	100.00
MgCO_3_ content	0.14	0.21	0.17	30.76	34.02	43.68	42.77	42.42

On the basis of the results, it was possible to determine a high magnesium calcite chemical formula of upper Biohermal limestone: (Ca_0.78–0.77_,Mg_0.22–0.23_)CO_3_.

On the basis of microprobe measurement results it can be concluded that three carbonate phases, different in magnesium content, built Triassic limestone of the Polish part of the Germanic Basin. The first one is low magnesium calcite with the content of Mg of about 1.81% (6.33% of MgCO_3_). The second phase is high-Mg calcite with the content of Mg changing from about 3.92–9.65% (MgCO_3_: from 13.72 to 33.77%). The third one is the dolomite phase which is characterized by varying content of MgCO_3_, from very low amounts of about 35.88–44.33% of MgCO_3_. These values are lower than the stoichiometric value for dolomite 46.13% MgCO_3_ (13.18% Mg). Therefore, this mineral phase is recognized as protodolomite.

The results of microprobe measurements confirmed the hypothesis of the presence of high-Mg calcite in studied Triassic limestones. On the basis of these results it was possible to determine the variability of Mg content in high-Mg calcite crystals. The results of the microprobe measurements indicate that beside of echinoderms the high-Mg calcite is also observed in other bioclasts. However, this phase most often built micritic cement (Fig. 5A, C).

#### Mapping executed with the use of the microprobe measurements method

Mapping was executed to differentiate the types of high-Mg calcite and changes in the magnesium content inside single crystals. The analysis was carried out with the use of the EDS method. Measurements were executed in carbonate ground mass. The results of mapping show that three carbonate phases are present in the investigated samples: low magnesium calcite, high-Mg calcite and dolomite. It confirms the results of the microprobe measurements carried out using the WDS method.

**Fig. 6:**
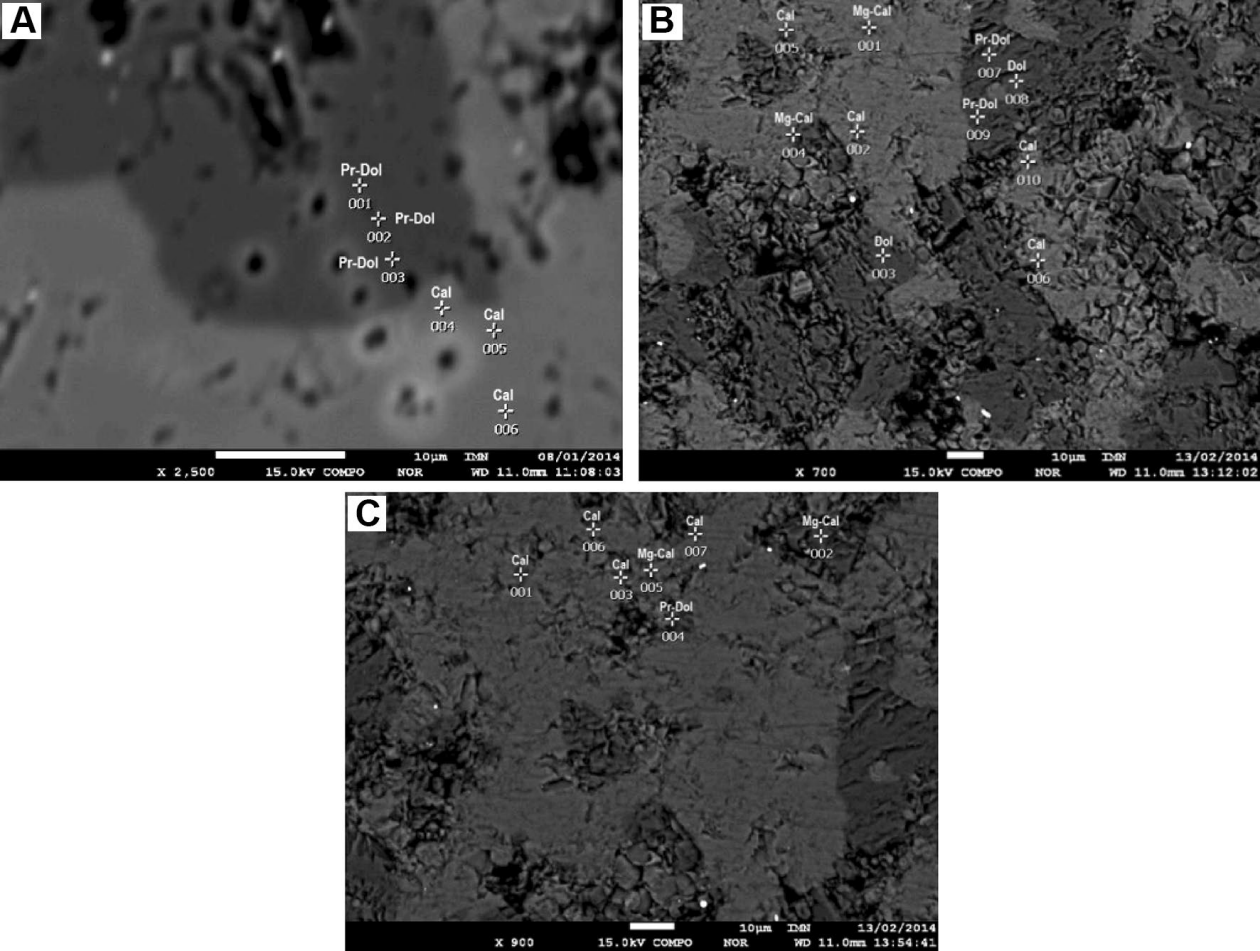
**A** BSE image of an lower Crinoidea limestone (sample 7). Magn. ×2500, 1–6-points of chemical analysis. Cal—low-Mg calcite; Pr-Dol—protodolomite. **B** BSE image of an upper Biohermal limestone (sample 41). Magn. ×700, 1–10-points of chemical analysis. Cal—low-Mg calcite; Mg-Cal—high-Mg calcite; Pr-Dol—protodolomite; Dol—ordered dolomite. C. BSE image of an Upper Biohermal Limestone (sample 41). Magn. ×900, 1–7-points of chemical analysis. Cal—low-Mg calcite; Mg-Cal—high-Mg calcite; Pr-Dol—protodolomite

In the microarea of lower Crinoidea limestone (sample 7), the analysis was executed in six points (Fig. 6A, Table 7). Three points (1, 2, and 3) were localized in the dark grey crystal of the carbonate phase rich in magnesium and other three points (4, 5 and 6) in the zone of fair grey rock mass built of carbonate phase and characterized by low content of magnesium (Fig. 6A).

**Table 7:** Microprobe chemical analyses in the microarea of the lower Crinoidea limestone (sample 7)

Chemical elements	Number of analyzed points according to Fig. 15 [% mass.]					
	1 Protodolomite	2 Protodolomite	3 Protodolomite	4 Low-Mg calcite	5 Low-Mg calcite	6 Low-Mg calcite
C	10.10	9.80	8.90	11.60	10.00	10.20
Mg	12.30	12.10	12.70	0.90	0.30	0.10
Ca	24.00	23.60	24.20	38.80	41.50	40.80
Fe	0.60	0.60	0.60	0.10	0.10	0.00
O	53.00	53.90	53.60	48.60	48.10	48.90
Total	100.00	100.00	100.00	100.00	100.00	100.00
MgCO_3_ content	43.05	42.35	44.45	3.15	1.05	0.35

The results allowed to show that the dark grey crystals represent dolomite. The analyses in points 1, 2 and 3 were executed inside a single crystal. There are no great differences in Ca and Mg content in the crystal of the dolomite phase (the amount of Ca ranges from 23.60 to 24.20% and Mg: from 12.10 to 12.70%) (Table 7). However, the content of Mg in this phase is lower than the stoichiometric value for dolomite—13.18% (46.13% of MgCO_3_). It indicates the presence of a dolomite phase rich in Ca (probably protodolomite). The results of the measurements carried out in points 4, 5 and 6 show that fair grey rock mass is built of low-Mg calcite. The amount of Ca in the low magnesium calcite phase ranges from 38.80 to 41.50% and Mg: only from 0.10 to 0.90% (Table 7). The results of mapping executed in the microarea of lower Crinoidea limestone using microprobe measurements with application of the EDS method confirm the results of the microprobe measurement carried out with use of the WDS method.

In the first microarea of the upper Biohermal limestone (sample 41) the analysis was executed in ten points (Fig. 6B, Table 8). Six points (1, 2, 4, 5, 6 and 10) were localized in the fair grey rock mass and four inside the dark grey crystals of a carbonate phase rich in magnesium (Fig. 6B). Point 3 was localized in the center of a small euhedral crystal and points 7, 8 and 9 also inside of a single euhedral crystal, point 8—in the center of the crystal and points 7 and 9—near the edges of the rhombohedron. The results tests show that in points 2, 5, 6 and 10 low-Mg calcite was identified. The amount of Ca in the low-Mg calcite phase ranges from 26.20 to 38.20%, Mg: from 0.10 to 5.00%, C: from 11.50 to 13.50% and O: from 50.20 to 57.50% (Table 8). High-Mg calcite occurs in points 1 and 4. The amount of Ca in high-Mg calcite phase ranges from 18.20 to 33.60%, Mg: from 8.00 to 10.20%, C: from 11.10 to 15.00% and O: from 47.30 to 55.40% (Table 8). The calcite phases are mixed in the micritic rock mass so it was not possible to observe the changes in Mg content inside of the high magnesium calcite crystals. The amount of Ca in the dolomite phase ranges from 22.20 to 24.30%, Mg: from 12.20 to 13.30%, C: from 12.10 to 12.70% and O: from 53.00 to 53.90% (Table 8). The differences in Ca and Mg content in points 7, 8 and 9 were observed. These points were situated inside a single crystal. The value of magnesium content is particularly important. In point 8, situated in the centre of crystal, the value is 13.30% (46.55% of MgCO_3_). It indicates the presence of typical ordered dolomite. But the values of Mg in points 7 and 9 are lower than the stoichiometric value for dolomite. They are characteristic rather for protodolomite. Because of these results, it is very difficult to say if the crystal for which the analysis was carried out in points 7, 8 and 9 is protodolomite as characterized by varying content of magnesium inside the crystal or if it is dedolomite in which in the center of the crystal the value of Mg is almost stoichiometric for dolomite and in the edge zones it is lower, which was caused by reduction during the process of dedolomitization. According to the results of the microprobe measurements executed using the WDS method, it could be rather protodolomite as characterized by varying Mg content in different zones of the crystal.

**Table 8:** Microprobe chemical analyses in first microarea of the upper Biohermal limestone (sample 41)

Chemical elements	Number of analyzed points according to Fig. 16 [% mass.]									
	1 High-Mg calcite	2 Low-Mg calcite	3 Dolomite	4 High-Mg calcite	5 Low-Mg calcite	6 Low-Mg calcite	7 Protodolomite	8 Dolomite	9 Protodolomite	10 Low-Mg calcite
C	11.10	11.50	9.40	15.00	11.80	13.50	10.70	11.00	12.30	12.40
Mg	8.00	0.10	13.30	10.20	5.00	2.80	12.50	13.30	12.20	2.20
Ca	33.60	38.20	22.70	18.20	30.90	26.20	22.90	24.30	22.20	33.70
Fe	0.00	0.00	0.00	1.20	0.00	0.00	0.00	0.00	0.70	0.00
O	47.30	50.20	54.60	55.40	52.30	57.50	53.90	51.40	52.60	51.70
Total	100.00	100.00	100.00	100.00	100.00	100.00	100.00	100.00	100.00	100.00
MgCO_3_ content	28.00	0.35	46.55	35.70	17.50	9.80	43.75	46.55	42.70	7.70

In the second microarea of the upper Biohermal limestone (sample 41), the analysis was executed in seven points (Fig. 6C, Table 9). Four points (1, 3, 6 and 7) were localized in the fair grey rock mass and three inside dark grey crystals of a carbonate phase rich in magnesium (Fig. 6C). Point 2 was localized in the center of a bigger euhedral dark grey crystal and points 4 and 5 inside a single very small dark grey crystal. The results of the tests show that in points 1, 3, 6 and 7 low-Mg calcite was identified. The amount of Ca in low-Mg calcite phase ranges from 32.60 to 36.80%, Mg: from 2.70 to 4.40%, C: from 10.40 to 12.70% and O: from 46.70 to 51.50% (Table 9). High-Mg calcite occurs in points 2 and 5. The amount of Ca in the high-Mg calcite phase ranges from 29.10 to 29.40%, Mg: from 7.30 to 9.80%, C: from 7.90 to 12.00% and O: from 46.40 to 51.30% (Table 9). The high-Mg calcite identified in point 2 forms an euhedral crystal, rhombohedral in shape and the high-Mg calcite determined in point 5 forms micritic grains mixed with micritic grains of low-Mg calcite. Point 2 was probably localized inside a small high-Mg calcite inclusion, located in the centre of the dolomite crystal. It is also possible that this point was situated inside a protodolomite crystal. Like in the first microarea of upper Biohermal limestone, also in this one it is not possible to observe the distribution of Mg and the changes of content of this element inside a single high-Mg calcite crystal. Point 4 was situated inside a dolomite phase. The amount of Ca in the dolomite phase is 19.20%, Mg: from 11.00%, C: 12.70% and O: 56.10% (Table 9). The value of the Mg content in the dolomite phase indicates that in this microarea of upper Biohermal limestone protodolomite is also present.

**Table 9:** Microprobe chemical analyses in the second microarea of the upper Biohermal limestone (sample 41)

Chemical elements	Number of analyzed points according to Fig. 17 [% mass.]						
	1 Low-Mg calcite	2 High-Mg calcite	3 Low-Mg calcite	4 Protodolomite	5 High-Mg calcite	6 Low-Mg calcite	7 Low-Mg calcite
C	10.40	7.90	12.40	12.70	12.00	12.70	12.00
Mg	2.70	9.80	4.40	11.00	7.30	3.20	4.10
Ca	36.80	29.10	36.50	19.20	29.40	32.60	34.00
Fe	0.00	6.80	0.00	1.00	0.00	0.00	0.00
O	50.10	46.40	46.70	56.10	51.30	51.50	49.90
Total	100.00	100.00	100.00	100.00	100.00	100.00	100.00
MgCO_3_ content	9.45	34.30	15.40	38.50	25.55	11.20	14.35

#### ICP AES spectroscopy

ICP AES spectroscopy was executed mainly to establish median average contents of Ca and Mg elements in the investigated limestones, and the amounts of Fe, Mn, Sr and Ba elements (Table 10). The content of Ca ranges from 27.30% in upper Crinoidea limestone to 32.90% in upper Biohermal limestone, Mg: from 7.00% in lower Crinoidea limestone to 9.10% in upper Biohermal limestone, Fe: from 0.19% in upper Crinoidea limestone to 0.25% in lower Crinoidea limestone, Mn: from 430 ppm in lower Biohermal limestone to 550 ppm in upper Biohermal limestone, Sr: from 120 ppm in lower Biohermal limestone to 190 ppm in upper Biohermal limestone and Ba: from 105 ppm in lower Biohermal limestone to 170 ppm in upper Biohermal limestone. The results of the study show that the analyzed rocks are built of high-Mg calcite, which is further confirmed by high Fe content. Iron usually substitutes magnesium in carbonate minerals, which are rich in this element. The values of Mg content indicate the presence of high-Mg calcite in the limestones of the Polish part of the Germanic Basin. Probably manganese also substitutes other chemical elements in the carbonate phases. The presence of Sr and Ba in the carbonate phases indicates that the primary sediment consist of aragonite as well. This mineral was transformed into low-Mg calcite during a process of dissolution, when the micritic and palisade cements were formed (the eogenetic stage of diagenesis).

**Table 10:** Results of ICP AES analysis

Chemical elements	Number of analyzed samples			
	Sample 7 Lower Crinoidea limestone	Sample 1 Lower Biohermal limestone	Sample 22 Upper Crinoidea limestone	Sample 41 Upper Biohermal limestone
Ca (%)	31.50	29.40	27.30	32.90
Mg (%)	7.00	7.70	9.00	9.10
Fe (%)	0.25	0.20	0.19	0.22
Mn (ppm)	450	430	520	550
Sr (ppm)	135	120	175	190
Ba (ppm)	120	105	155	170

### Preservation conditions of the high-Mg calcite in the Muschelkalk sediments

#### High-Mg calcite in lower Muschelkalk of Opole Silesia

The results of the analyses allowed to show that in all formations of Muschelkalk high magnesium calcite is present apart from low-Mg calcite, dolomite, and smaller amounts of quartz and clay minerals.

The results of a microscopic analysis, microprobe measurements executed using the WDS method and a mapping carried out by microprobe measurements applying the EDS method allowed to show that small xenomorphic crystals of high-Mg calcite build the rock mass of bioclastic and organodetritic limestones together with low magnesium calcite. This rock mass could be micritic, microsparry or sparry. High-Mg calcite also forms small xenomorphic crystals in the micritic rock mass of dolomite limestone. Therefore, it was not possible to distinguish this carbonate phase from low-Mg calcite in the thin sections and in most of the BSE images. Only the microprobe measurements executed in some points confirmed the presence of high-Mg calcite in the investigated limestones. This unstable carbonate phase was also identified on the basis of the results of Fourier Transform Infrared Spectroscopy, X-ray diffraction and ICP OES Spectroscopy.

In infra-red absorption curves high magnesium calcite was identified on the basis of the *V*_3_ absorption band (values from 1428 to 1435), *V*_1_ + *V*_3_ absorption band (values from 2517 to 2521) and in some samples on the basis of other absorption bands like 2621, 2625, 2939, 2942, 3021 and 3470. The absorption bands of this carbonate phase have higher values than those typical for low magnesium calcite but lower than the ones prevalent for dolomite (Ahn et al. 1996; Böttcher et al. 1997; Ji et al. 2009; Pokrovsky et al. 2000). The results of a FTIR analysis allowed to indicate increased content of Mg in high magnesium calcite of upper Biohermal limestone in comparison with the carbonate phase of other limestone formations. The results of the FTIR analysis were confirmed by results of X-ray diffraction. High-Mg calcite diffraction lines observed in X-ray diffraction patterns show constants of the lattice lower in comparison to the typical ones for low magnesium calcite. For this reason high magnesium calcite was identified mainly on the basis of the following *d*_hkl_ values: 2.990 Å (*I* = 100), 2.266 Å (*I* = 15), 1.857 Å (*I* = 15), 1.423 Å (*I* = 3), 1.410 Å (*I* = 3), 1.193 Å (*I* = 3).

More details, connected especially with the chemical formula and the crystal structure of high-Mg calcite were obtained during microprobe measurements carried out using the WDS method and a mapping made with application of the EDS method. The results of the measurements allowed to show that high magnesium calcite of each limestone formation are characterized by different content of Mg. The lowest amount of magnesium in high-Mg calcite was observed in limestones of lower Crinoidea formation, which built the lower part of the lower Muschelkalk Profile. The highest amount is present in the limestones of Lower Biohermal Formation. The content of MgCO_3_ in high magnesium calcite of investigated rocks ranges in lower Crinoidea limestone—from 22.82 to 33.11%, in lower Biohermal limestone—from 29.61 to 36.89%, in upper Crinoidea limestone the value is 30.52% and in upper Biohermal limestone it ranges from 30.76 to 34.02%. According to the results of the microprobe measurements, the highest content of MgCO_3_ in low magnesium calcite has a value of 8.22% and the lowest amount of MgCO_3_ in protodolomite is 40.60%. Therefore, the amounts of MgCO_3_ in high-Mg calcites of the investigated limestones are higher than in low magnesium calcite but lower than in dolomite.

The median values of magnesium calculated for high-Mg calcite of the investigated limestones show that high magnesium calcite of Biohermal limestones generally contains higher amount of magnesium (median value-above 9% of Mg) than Crinoidea limestones (median value-below 9% of Mg). The content of MgCO_3_ calculated for the lowest amount of Mg: 6.52% of high-Mg calcite exceeds the value of 22.82%. In the case of the highest amount of Mg: 10.54% the calculated value of MgCO_3_ is 36.90%. The results of the microprobe measurements allowed to determine a high magnesium calcite chemical formula, which presents the possible range of magnesium content in crystal of high-Mg calcite. According to the results of microprobe measurements general chemical formulas of high magnesium calcite are as following: (Ca_0.87–0.80_,Mg_0.13–0.20_)CO_3_ for lower Crinoidea limestone, (Ca_0.83–0.75_,Mg_0.17–0.25_)CO_3_ for lower Biohermal limestone, (Ca_0.81_,Mg_0.19_)CO_3_ for upper Crinoidea limestone and (Ca_0.78–0.77_,Mg_0.22–0.23_)CO_3_ for upper Biohermal limestone. The chemical formulas show that high magnesium calcites of Biohermal limestones generally contain higher amounts of magnesium than high-Mg calcites of Crinoidea limestones. The content of MgCO_3_ of high-Mg calcite exceeds the value of 22.7%. The results of the microprobe measurements executed using the WDS method were confirmed by the data obtained by mapping. The results of the mapping also allowed to indicate that micritic grains of high-Mg calcite are mixed with micritic grains of low-Mg calcite. These two carbonate phases build the limestone rock mass. Moreover, the results confirmed the occurrence of protodolomite in the investigated rocks and, subsequently the theory of a primary origin of high-Mg calcite and protodolomite. The results of mapping executed by microprobe measurements with application of EDS method allowed to show that high magnesium calcite forms micritic grains which are mixed with micritic grains of low-Mg calcite. Thus, it was not possible to analyze the changes of magnesium content inside a single crystal of this carbonate phase. The changes of Mg amount could be observed only in crystals of dolomite phase. The amount of Ca in high-Mg calcite phase ranges from 18.20 to 33.60%, Mg: from 7.30 to 10.20%, C: from 7.90 to 15.00% and O: from 46.40 to 55.40%. But low content of magnesium in dolomite phase in comparison with stoichiometric value for dolomite: 13.18% (46.13% of MgCO_3_) and the lack of big changes of Mg inside of single dolomite crystals seem to be of more importance here. These indicate the presence of protodolomite in the investigated limestones. This non-stoichiometric, poorly ordered carbonate phase which forms euhedral and rhombohedral-shaped crystals is usually created in shallow epicontinental seas during early stages of diagenesis (Boggs 2010; Morse and Mackenzie 1990; Tucker and Wright 1990).

The results of all executed analyses allowed to indicate the presence of high-Mg calcite in the investigated limestones. It was important to compare the results of all analyses, especially results of the X-ray diffraction and the microprobe measurements. According to Zhang et al. (2010), with the increase of MgCO_3_ content in high magnesium calcite, the values of cell parameters—*a*_0_ (Å) and *c*_0_ (Å) drop. Analyzing data obtained by Zhang et al. (2010), it is possible to determine cell parameters on the basis of MgCO_3_ content. When the amount of MgCO_3_ is 22.7%, the cell parameters of high-Mg calcite present general values as follows: *a*_0_ = 4.91 Å, *c*_0_ = 16.65 Å and for the amount of MgCO_3_ 36.71% the cell parameters present general values: *a*_0_ = 4.88 Å, *c*_0_ = 16.45 Å (Zhang et al. 2010). On the basis of the peak values, mainly of *d*_104_, visible in the X-ray diffraction patterns of investigated limestones, *a*_0_ and *c*_0_, the parameters established according to the ICDD Card Index have the following values: *a*_0_ = 4941 Å, *c*_0_ = 16,854 Å. These values, similar to the parameters established by Johnson (2002) (*a*_0_ = 4.942 Å and *c*_0_ = 16.85 Å) for 47.6% of MgCO_3_, indicate the presence of an increased amount of magnesium in high-Mg calcite. However, these values were not calculated but established only on the basis of the data from the ICDD Card Index. The results of the microprobe measurements show that the amount of MgCO_3_ in high magnesium calcite varies from 22.82 to 36.89%. It indicates the different content of magnesium in high-Mg calcite. This analysis was executed in individual points, so the differences could be observed not only in calcium and magnesium contents but also in iron content. Generally speaking higher amounts of iron were observed in the carbonate phases showing also increased amounts of magnesium. It is probably connected with the possibility of magnesium being substituted by iron in the carbonate phases rich in magnesium. Some factors influence the substitution. The most important are: cationic size, cationic radius, M–O bond length, cell volumes, correlation between M–O bond length and *c*/*a* ratios (Tucker and Wright 1990). Fe could substitute Mg because these two chemical elements have similar cationic size and length of the cationic radius.

As a result of magnesium substitutions in the crystals of high-Mg calcite, the structure of crystal cells is different in comparison with a low magnesium calcite crystal. It is connected with a difference in the size of the ionic radius of Ca and the ionic radius of Mg (Titiloye et al. 1998; Tsipursky and Buseck 1993). Ca and Mg ions take the positions in space network alternately along the threefold axis; therefore the symmetry of calcite is reduced. Instead of a scalenohedral ditrigonal symmetry, which includes 3L^2^ (three twofold axes) apart from other symmetry parameters, a rhombohedral symmetry similar to the symmetry of a dolomite crystal structure occurs. It can be observed in carbonate minerals rich in magnesium, such as: protodolomite (Ca_0.5_,Mg_0.5_CO_3_; space group R3c), ordered dolomite (Ca_0.5_,Mg_0.5_CO_3_; space group R3), huntite (Ca_0.25_,Mg_0.75_CO_3_; space group R32) and magnesite (MgCO_3_; space group R3c) (Böttcher et al. 1997). The symmetry of a high magnesium calcite crystal is rhombohedral with the space group R3c (Althoff 1977; Paquette and Reeder 1990), similar to the one typical for protodolomite.

#### The theory on the preservation conditions of high-Mg calcite

The results of the study allowed to show that the highest content of Mg in high magnesium calcite characterizes rocks of lower Crinoidea Beds and the lowest-rocks of upper Biohermal Beds, so the content of Mg decrease from the bottom to the top of analyzed profile. This falling trend of Mg towards the top of the section is indicative of diagnenetic processes influence. Meteoric water induced depletion of Mg. Karchowice Beds represent barrier sediments of reef facies (Bodzioch 2005). Their formation was connected with the beginning of the sea regression. The sea-level fall could cause a progressive increase of salinity. Normally, the high-Mg calcite is transformed into low-Mg calcite with increasing burial depth and low magnesium calcites dominate in the ancient limestones. Aragonite and high-Mg calcite are the dominant carbonate phases in warm tropical to subtropical waters. In cooler waters and in the depth of the ocean, the Mg content decrease and low magnesium calcite dominates (Mackenzie and Andersson 2013). The temperature and the Mg/Ca ratio are factors increased the probability of dolomite and calcites rich in Mg formation and high-Mg calcite preservation (Morse and Mackenzie 1990). When the temperature of seawater is typical for tropical seas (25–30 °C) but the Mg/Ca ratio is below 1, low magnesium calcite precipitates. When Mg/Ca ratio is above 1 at normal temperatures, high magnesium calcite precipitates. These conditions could also cause a poorly ordered protodolomite formation (Boggs 2010). The content of Mg increases with the increase of Mg/Ca ratio depending on temperature (Stanley et al. 2002; Stanley 2008). At temperatures over 42 °C, high-Mg calcite with the content of up to 15 mol% of MgCO_3_ is stable in relation to low-Mg calcite (Bertram et al. 1991). The calculated temperatures of low-Mg calcite crystallization (Stanienda 2011, 2013) exceeds the value of 42 °C. For lower Crinoidea limestone it is 59.66 °C, for upper Crinoidea limestone—from 61.10 to 65.97 °C and for Upper Biohermal Limestone—from 58.82 to 76.29 °C. Crystallization temperatures of high-Mg calcite present values: for upper Crinoidea limestone—from 49.94 to 53.71 °C and for Upper Biohermal Limestone—64.91 °C (Stanienda 2013). They are not normal temperatures even for tropical seawaters. Therefore, the results can suggest the influence of diagenetic processes on high-Mg calcite preservation, because this carbonate phase could be preserved in higher temperatures. Diagenesis started after the deposition of sediment. However, some diagenetic processes started before sediment compaction. The temperature increase is connected with burial depth. Preservation of high-Mg calcite depends also on different other factors such as Mg/Ca ratio, solubility, calcium and magnesium ion activity, the type of environment, as well as on the influence of marine organisms. Based on Mg content of well preserved Triassic echinoderms, Dickson (2004), estimated that the Triassic seawater Mg/Ca ratio had a value of about 3.4. Nevertheless, it has been recently argued that application of fossil echinoderms to seawater Mg/Ca reconstructions is unreliable (Gorzelak et al. 2013, 2016). As inferred from the Hardie (1996) curve, Mg/Ca ratio of Triassic seawater was slightly lower; it ranged from about 2.5–3. Therefore, also the conditions of seawater (probably tropical shallow sea), especially higher salinity along with value of Mg/Ca ratio of seawater connected with the shallowing of the sea basin, increased content of Na (Stanienda 2000, 2011, 2013), CO_2_, pressure and also neomorphic processes (Boggs 2010) could influence the preservation of high magnesium calcite in Muschelkalk limestones of the Polish part of the Germanic Basin. The general effect of diagenesis, especially in vadose zone, is a destruction of less-stable aragonite and low magnesium calcite and concomitantly generating stable high-Mg calcite (Boggs 2010). Therefore, the most probable is the influence of diagenetic processes—a marine water drainage, increase of temperature and of other seawater—factors (increased CO_2_, increased salinity, pressure) also neomorphism which could finally caused the preservation of high-Mg calcite.

#### Condition and environment of high-Mg calcite formation

Analyzing the possibilities of high-Mg calcite forming, the carbonate phase is characterized by a higher than in low magnesium calcite but lower than in the protodolomite amount of magnesium. This mineral was created in a shallow sea, in an environment of warm, salty seawater, inside a carbonate sediment during its compaction (early stage of diagenesis). The analyzed rocks, which contain high-Mg calcite, were formed in the eastern part of the Germanic Basin, in a shallow zone during the beginning of the sea regression. The salinity of the water was probably increasing because of the shallowing of the sea basin. The variety of chemical elements indicates the increased salinity of seawater in which high magnesium calcite was formed. While analyzing the possibility of the influence of dolomitization on the high-Mg calcite formation it is necessary to say that dolomitization of aragonite proceeds faster than dolomitization of calcite (Morse and Mackenzie 1990). High magnesium calcite may or may not be dolomitized faster than low magnesium calcite (Morse and Mackenzie 1990). It depends of different factors: solubility, Mg/Ca ratio, temperature, Ca and Mg ion activity. In case of the investigated Crinoid limestones, some amounts of magnesium could come from marine organisms: shells and different parts of skeletons, which had an elevated level of high-Mg calcite. It was possible to observe crinoid circular stem plates both with the naked eye and during the microscopic analysis. Some of them are built of micritic calcite, others of single calcite crystals, with a rhombohedral cleavage visible in some examples. Micritic calcite, which forms stem plates, could be the primary calcite phase. However, while studying thin sections, it was impossible to distinguish between low-Mg calcite and high-Mg-calcite. The single calcite crystals with a visible cleavage, which built stem plates, are probably the second generation calcite crystals which could be formed by a transformation of the primary carbonate phases during the diagenetic processes. After the carbonate material deposition during the shallowing of the sea basin, the compaction processes began (early stage of diagenesis-eogenetic stage). It was characterized by a reduction of water in sediment and a concentration of chemical components. It also caused an increase of salinity of the carbonate material what could also influence the preservation of high magnesium calcite. Effects of different types of diagenesis were observed in the studied limestones. Destructive diagenesis is mainly connected with the activity of marine organisms, which changed the surface of bioclasts by burrowing. Burrows were filled with microcrystalline calcium carbonate-aragonite or high-Mg calcite. The presence of Sr and Ba in the carbonate phases indicates that the primary sediment also included aragonite which was transformed into low-Mg calcite during the process of dissolution, when the micritic and palisade cements were formed (the eogenetic stage of diagenesis). During the eogenetic stage of diagenesis high-Mg calcite and dolomite were created. According to Boggs (2010), Fairbridge (1957), Flügel (2004) and Szulc (1990, 2000), skeletons of marine fauna are other source of magnesium (skeletons of echinoderms, coralline algae, foraminifers). Echinoderms, especially crinoids dominate in analyzed rocks. During the mesogenetic stage of diagenesis, the mineral phases of carbonate sediment underwent aggradation, recrystallization, dissolution and transformation processes. Dissolution processes were taking place under pressure in reductive environment. Iron, which replaced magnesium in carbonate crystals, was released from these crystals and filled stylolites, which were formed during the dissolution of carbonates. Analyzing the tests results and the type of the environment in which diagenetic processes, which allowed for high-Mg calcite formation, take place (Tucker and Wright 1990) and using the Boggs (2010) division, it can be assumed that the diagenetic processes in limestones of the Polish part of the Germanic Basin could probably occur in the following zones: in the deep sea zone-phreatic sea zone and in the zone of the shallow sea floor and shallow subsurface areas). In some areas—in meteoric zone.

## Conclusions

The principal results can be summarized as follows:

1. The results of the executed analyses allowed to confirm the hypothesis of the high magnesium calcite presence in Karchowice Beds of the area of Polish part of the Germanic Basin.
2. Micritic grains of high-Mg calcite are mixed with micritic grains of low magnesium calcite. These two carbonate phases built the rock mass in bioclastic and organodetritic limestones.
3. The results of the microprobe measurements allowed to show that, the amount of MgCO_3_ in high magnesium calcite ranges from 22.82 to 36.89% and it ranges in lower Crinoidea limestone—from 22.82 to 33.11%, in lower Biohermal limestone—from 29.61 to 36.89%, in upper Crinoidea limestone the value is 30.52% and in upper Biohermal limestone it ranges from 30.76 to 34.02%. These values are higher than the highest calculated value for low-Mg calcite—8.22% of MgCO_3_ and the lowest value calculated for protodolomite—40.60% of MgCO_3_.
4. The results of the microprobe measurements, allowed to determine the high magnesium calcite general chemical formula, which presents a possible range of the magnesium content in the crystals of high-Mg calcite. High-Mg calcite of lower Crinoidea limestone has a chemical formula as follows: (Ca_0.87–0.80_,Mg_0.13–0.20_)CO_3_. A general chemical formula of high magnesium calcite of lower Biohermal limestone is as follows: (Ca_0.83–0.75_,Mg_0.17–0.25_)CO_3_, of upper Crinoidea limestone is (Ca_0.81_,Mg_0.19_)CO_3_ and the formula of upper Biohermal limestone is (Ca_0.78–0.77_,Mg_0.22–0.23_)CO_3_.
5. On the basis of the peak values, mainly of *d*_104_, visible in the X-ray diffraction patterns of samples, according to the ICDD Card Index, *a*_0_ and *c*_0_ parameters were established. The values are: *a*_0_ = 4941 Å, *c*_0_ = 16,854 Å. These data confirm the presence of high-Mg calcite. The form of high-Mg calcite-micritic grains and the occurrence of protodolomite in the investigated limestones which present a similar content of Mg in different points inside of a single crystal, confirm the theory of the secondary, early diagenetic origin of this unstable carbonate phase and the same origin of protodolomite. High-Mg calcite was formed in the epicontinental Germanic Basin, in the environment of warm, salty sea water, inside a carbonate sediment during its compaction (early stage of diagenesis-eogenetic), at the same time as protodolomite. However, some amount of Mg-calcite, which was part of marine organism could build together with aragonite the primary sediment. The conditions of seawater, especially higher salinity and also advanced diagenetic processes including neomorphism influenced the preservation of high magnesium calcite, unstable carbonate phase, in Muschelkalk limestones of the Polish part of the Germanic Basin.
